# Dual-targeting VEGF and TNF-α with bispecific antibody eyedrops for synergistic treatment of uveitis

**DOI:** 10.1186/s12951-026-04681-y

**Published:** 2026-06-16

**Authors:** Jie Zhou, Yinqi Chen, Ruochu Guan, Wenchao Cai, Zhiqing Lin, Panxiang Rao, Sisi Chen, Penfeng Gao, Qian Yang, Ying Wu, Yihua Zhu, Biting Zhou, Nanwen Zhang, Juhua Yang, Xiaole Chen

**Affiliations:** 1 Department of Bioengineering and Biopharmaceutics, School of Pharmacy, Fujian Medical University, Fuzhou, 350122 Fujian China; 2 Fujian Key Laboratory of Drug Target Discovery and Structural and Functional Research, School of Pharmacy, Fujian Medical University, Fuzhou, 350122 Fujian China; 3 Department of Ophthalmology, The First Affiliated Hospital of Fujian Medical University, Fuzhou, 350000 Fujian China; 4 National Regional Medical Center, Binhai Campus of the First Affiliated Hospital, Fujian Medical University, Fuzhou, 350000 China; 5 Key Laboratory of Natural Medicine Pharmacology in Fujian Province, School of Pharmacy, Fujian Medical University, Fuzhou, 350122 Fujian China; 6 Department of Pharmacology, School of Pharmacy, Fujian Medical University, Fuzhou, 350122 Fujian China; 7 FuZhou Aier Eye Hospital, Fuzhou, 350000 Fujian China

**Keywords:** Uveitis, Anti-VEGF, Anti-TNF-α, Bispecific antibody, Thiol-mediated transport pathway

## Abstract

Biologics have emerged as frontline therapies for uveitis due to their targeted immunomodulatory effects. However, conventional monoclonal antibodies remain confined to single-target engagement. In addition, intravitreal injections carry risks of complications associated with repeated administration, whereas noninvasive delivery is hindered by compromised bioavailability across the complex ocular barriers. Together, these limitations restrict the effectiveness of current therapeutic approaches. Herein, bioinformatic screening identified VEGF and tumor necrosis factor alpha (TNF-α) as core pathogenic mediators in uveitis. Subsequently, we engineered a VEGF/TNF-α bispecific antibody (V5-3) and assembled it with a dithiolane molecule (DM) into nanoparticles (V5-3-NPs), thereby creating a noninvasive topical nanoformulation for uveitis therapy. Compared with V5-3, V5-3-NPs significantly enhanced cellular uptake, inhibited microglial proliferation and migration, and reduced the release of pro-inflammatory cytokines. In both in vitro and in vivo models, the synergistic bispecific targeting of V5-3-NPs enhanced anti-inflammatory and anti-angiogenic efficacy, thereby effectively alleviating clinical manifestations and histopathological damage. The negligible toxicity observed across models confirmed its safety profile and supported clinical feasibility. This work tackles key unmet needs in uveitis therapy by overcoming inefficient drug delivery and single-target constraints. By integrating bispecific targeting with nanocarrier-based delivery, it offers a distinct technological advance and a clinically viable strategy for uveitis treatment.

**Graphical Abstract:**

**Figure:**
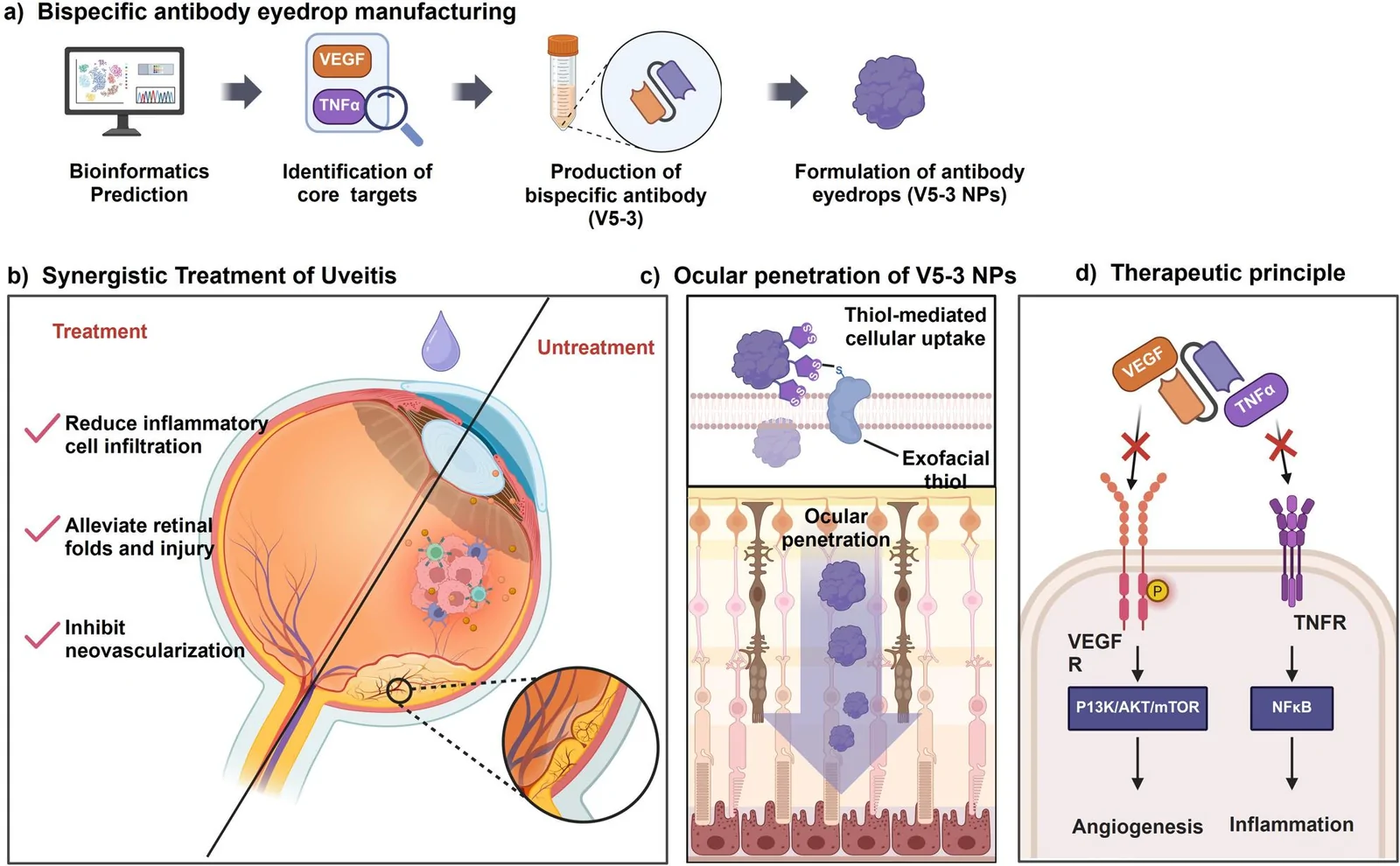
A VEGF/TNF-α bispecific antibody (V5-3) was engineered and assembled with a dithiolane molecule to form nanoparticles (V5-3-NPs) for topical administration. V5-3-NPs synergistically suppressed microglial activation, reduced inflammatory cytokines and neovascularization, and markedly alleviated retinal damage without detectable toxicity. This dual-target nano-biologic eyedrop offers a noninvasive and clinically promising strategy for uveitis therapy.

**Supplementary Information:**

The online version contains supplementary material available at 10.1186/s12951-026-04681-y.

## Introduction

Uveitis is a severe inflammatory disorder with a complex pathogenesis, primarily affecting the uvea and surrounding ocular structures. As one of the leading causes of blindness worldwide, uveitis poses a substantial threat to vision [1]. Clinical studies have reported that, even with specialized care, 20**–**70% of patients experience severe vision loss, up to 50% develop permanent visual impairment, and 10**–**15% progress to complete blindness [2].

Current uveitis management relies on corticosteroids, immunosuppressants, and biologics [3]. Although corticosteroids effectively suppress intraocular inflammation, their clinical utility is constrained by an unfavorable risk–benefit balance, as prolonged use can cause significant ocular and systemic complications [4]. Immunosuppressants are hindered by the delayed onset of action and risks of organ toxicity, requiring close clinical monitoring [5]. Although biologics (e.g., anti-TNF-α or anti-VEGF agents) provide targeted immunomodulation, their single-target mechanisms often lead to drug resistance and reduce clinical efficacy over time [6]. Thus, by simultaneously engaging dual targets, bispecific antibodies overcome the limitations of monospecific therapies and have emerged as a leading-edge therapeutic strategy with demonstrated efficacy in multiple uveitis studies [7, 8].

Topical eyedrops represent the most common treatment for ocular diseases. However, their efficacy is greatly limited by ocular anatomical and physiological barriers, which restrict drug penetration into deeper pathological tissues. Alternative delivery routes, including periocular or intraocular injections, provide more direct access and prolonged drug activity but are associated with pain, infection, and hemorrhagic risks. Systemic administration is widely practiced but remains largely ineffective due to the restrictive blood-retinal barrier [9, 10]. Nanotechnology-based delivery systems have emerged as a promising solution to these challenges. Engineered nanoparticles can selectively target specific ocular tissues or cell types, protect therapeutic agents from degradation, enhance absorption and bioavailability, and enable sustained release, thereby reducing dosing frequency and improving patient adherence. In uveitis therapy, most efforts have focused on small-molecule nanomedicines [11, 12]. For instance, multifunctional hydrogel-based eyedrops combining dexamethasone (DSP) and cerium-based metal–organic frameworks (Ce-MOFs) have shown synergistic anti-inflammatory and antioxidant effects, while ROS-responsive nanoparticles co-loaded with DSP and curcumin achieved superior efficacy in rabbit uveitis models at lower corticosteroid doses compared with free drugs or commercial eyedrops [13, 14]. Despite these advances, the development of noninvasive formulations for macromolecular biologics remains highly challenging. In particular, antibody-based eyedrops have not yet been reported for uveitis, underscoring a critical unmet need and highlighting the potential of innovative nanocarrier strategies to enable effective, noninvasive biologic therapies.

**Fig. 1:**
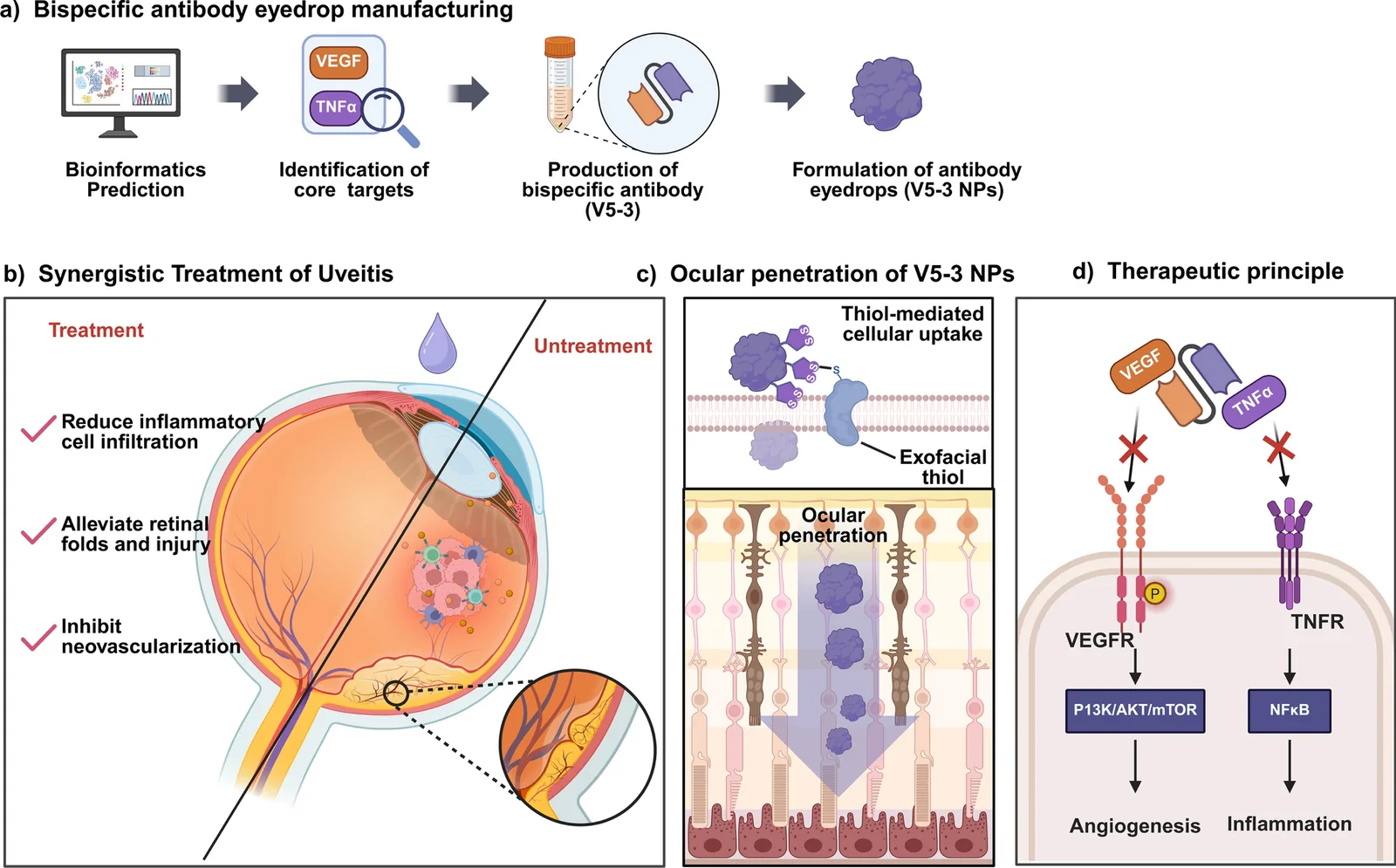
Schematic illustration of the manufacture, ocular penetration, and therapeutic mechanism of the bispecific antibody eyedrops (V5-3 NPs)

Previous studies have shown that nano-delivery systems incorporating dithiolane can efficiently penetrate compact tissues via a thiol-mediated transport mechanism, thereby enhancing the intraocular penetration of nucleic acids and antibodies. Herein, we identified a bispecific antibody and incorporated a DM to construct a nano-antibody eyedrop, aiming to overcome both the limitations of monospecific antibody therapies and the challenges of ocular tissue penetration (Fig. 1). Through bioinformatic analysis of uveitis-related targets, we identified key genes and pathways driving disease progression, with TNF, VEGFA, and IL-6 emerging as core pathogenic mediators. A bispecific antibody targeting TNF-α and VEGF (V5-3) was developed and demonstrated synergistic therapeutic efficacy by significantly inhibiting both angiogenesis and inflammation. We fabricated V5-3 nano-eyedrops (V5-3-NPs) using DM as carriers. Through noninvasive eyedrop administration, V5-3-NPs markedly alleviated both clinical symptoms and histopathological changes in experimental autoimmune uveitis (EAU) rats. Notably, the therapeutic efficacy of V5-3-NPs surpassed that of the commercial Pranoprofen eyedrops. Collectively, this innovative bispecific antibody eyedrop provides a promising and distinct strategy to enhance both the efficacy and safety of uveitis management.

## Results and discussion

### Bioinformatic prediction of uveitis-driving genes and pathways

**Fig. 2:**
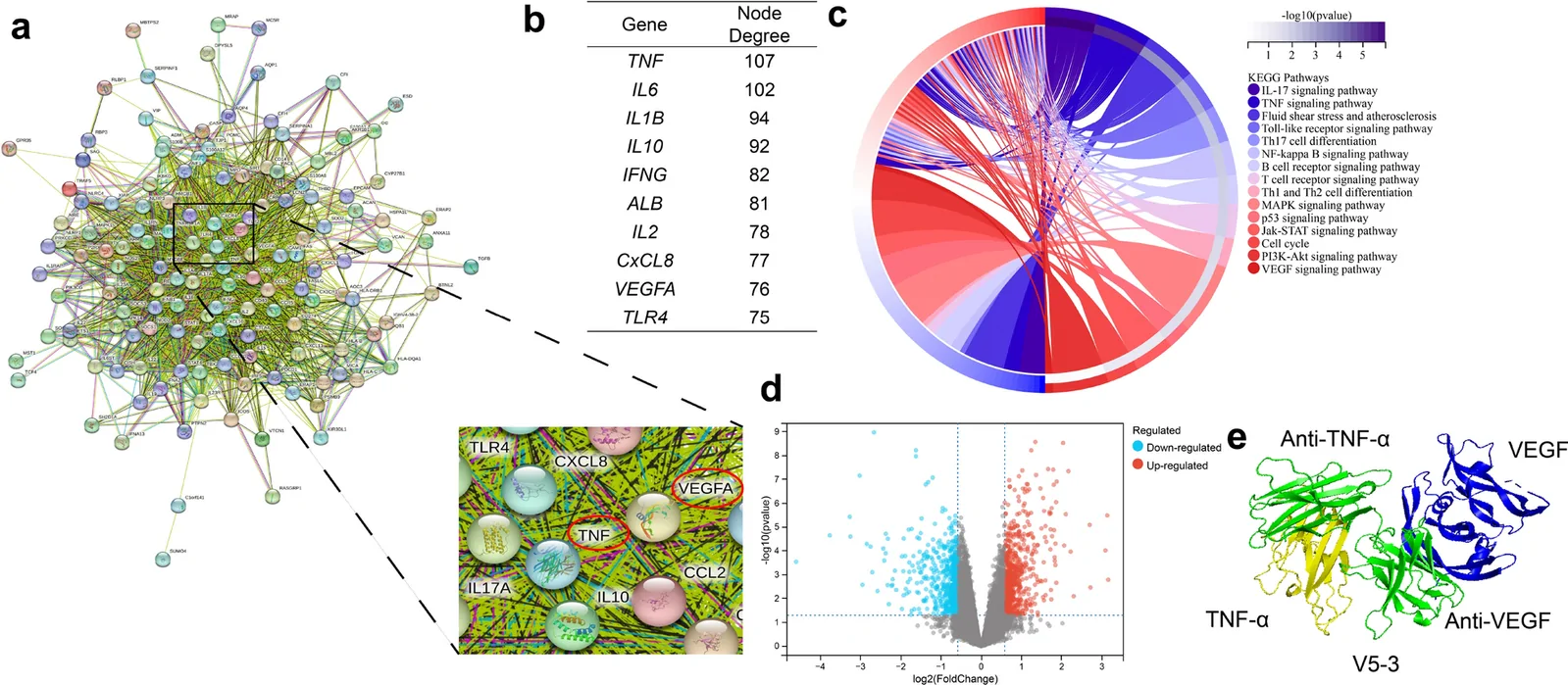
In Silico Design and Experimental Validation of V5-3 for Uveitis. (**a**) Protein–protein interaction (PPI) network of overlapping genes generated via STRING. Nodes represent genes with node size scaled to degree centrality. Edges indicate interactions with edge thickness proportional to confidence scores. (**b**) Top 10 hub proteins ranked by interaction score. (**c**–**d**) Identification of uveitis-associated differentially expressed genes (DEGs) through transcriptomic analysis. KEGG pathway analysis presented as a chord plot (**g**). Genes are shown on the left, and significantly enriched pathways are displayed on the right. Connections indicate gene enrichment within the corresponding pathways. Volcano plot of DEGs (**d**). Red: up-regulated genes, Blue: down-regulated genes. The x-axis represents log₂ fold change (log₂FC); genes farther from the origin exhibit larger absolute fold-change values. (**e**) Predicted 3D structure of V5-3 and molecular docking simulations with VEGF and TNF-α using Swiss-Model

The pathogenic genes associated with uveitis were retrieved from the DisGeNET and GeneCards databases. A total of 155 overlapping pathogenic genes were identified, as shown in the Venn diagram (Figure S1a). A protein–protein interaction (PPI) network of the identified targets was constructed using the Search Tool for the Retrieval of Interacting Genes/Proteins (STRING) database and visualized with Cytoscape (Fig. 2a). Using an interaction score threshold of > 0.4, the top 10 genes were screened as key node targets for uveitis (Fig. 2b). Among these, the top 5 genes, ranked by three Cyto-Hubba algorithms (Betweenness, BottleNeck, and Stress) with degree ≥ 1, were identified as hub genes (Figure S1b). Finally, Venn analysis identified TNF, VEGFA, and IL-6 as the core pathogenic genes of uveitis (Figure S1c).

Subsequently, we performed data mining by integrating differentially expressed genes (DEGs) obtained from the Gene Expression Omnibus (GEO) database (GSE66936), comparing samples from uveitis patients with healthy controls. Kyoto Encyclopedia of Genes and Genomes (KEGG) pathway analysis revealed that the DEGs were predominantly concentrated in the NF-κB, MAPK, Jak-STAT, PI3K-Akt, and VEGF signaling pathways (Figs. 2c and S1e). In total, 1267 mRNAs were identified, including 685 upregulated and 582 downregulated transcripts (Fig. 2d). Functional enrichment and signaling pathway analyses were further conducted to evaluate these targets using the Gene Ontology (GO) and KEGG databases. GO biological pathway (BP) analysis revealed that the DEGs were primarily enriched in cellular response to chemical stimulus, immune system processes, and organic substances. GO cellular components (CC) analysis showed enrichment in the cytosol, endomembrane system, and intracellular vesicles. GO molecular function (MF) analysis indicated enrichment in RNA binding, identical protein binding, and protein homodimerization activity (Figures S1d). KEGG pathway analysis revealed that the DEGs were predominantly concentrated in the NF-κB, MAPK, Jak-STAT, PI3K-Akt, and VEGF signaling pathways (Figs. 2c and S1e). These pathways delineate the pathogenic connections between uveitis progression and immune responses, providing critical insights for the subsequent study.

In uveitis, inflammation and angiogenesis form a mutually reinforcing pathological cycle. TNF-α primarily drives inflammatory cascades through TNFR1-mediated activation of the NF-κB and MAPK (JNK and p38) signaling pathways, leading to the transcriptional upregulation of multiple pro-inflammatory mediators, including IL-6, ICAM-1, and COX-2 [15, 16]. Concurrently, VEGF binds to VEGFR2 and activates the MAPK/ERK and PI3K/Akt pathways, thereby promoting endothelial cell proliferation, angiogenic sprouting, vascular leakage, and the activation and recruitment of inflammatory cells [17]. Importantly, these signaling pathways do not function independently but exhibit extensive crosstalk at multiple regulatory nodes. TNF-α can induce VEGF expression mainly through NF-κB signaling, with additional contributions from the PI3K/Akt, MAPK, and STAT3 pathways. Conversely, VEGF further amplifies inflammatory responses by facilitating inflammatory cell infiltration and enhancing the production of pro-inflammatory cytokines [18]. Therefore, we hypothesize that a bispecific antibody simultaneously targeting these interconnected inflammatory and angiogenic pathways may achieve superior therapeutic efficacy in uveitis [17].

### Construction of bispecific antibody targeting VEGF/TNF-α

In light of our earlier identification of TNF and VEGFA as key genes in uveitis, the bispecific antibody targeting VEGF/TNFα (V5-3) was constructed with our reported methods [19]. The 3D protein structure models of V5-3 were then predicted using SWISS-MODEL. The PDB data of TNF-α and VEGFA were retrieved from the RCSB database. The binding mechanisms and potential docking interactions between TNF-α, VEGF, and V5-3 were simulated by HawkDock. The 3D structures were visualized with PyMOL software (Fig. 2e). These results suggested that V5-3 could specifically bind to VEGF and TNF-α, which was further supported by subsequent enzyme-linked immunosorbent assay (ELISA) results.

**Fig. 3:**
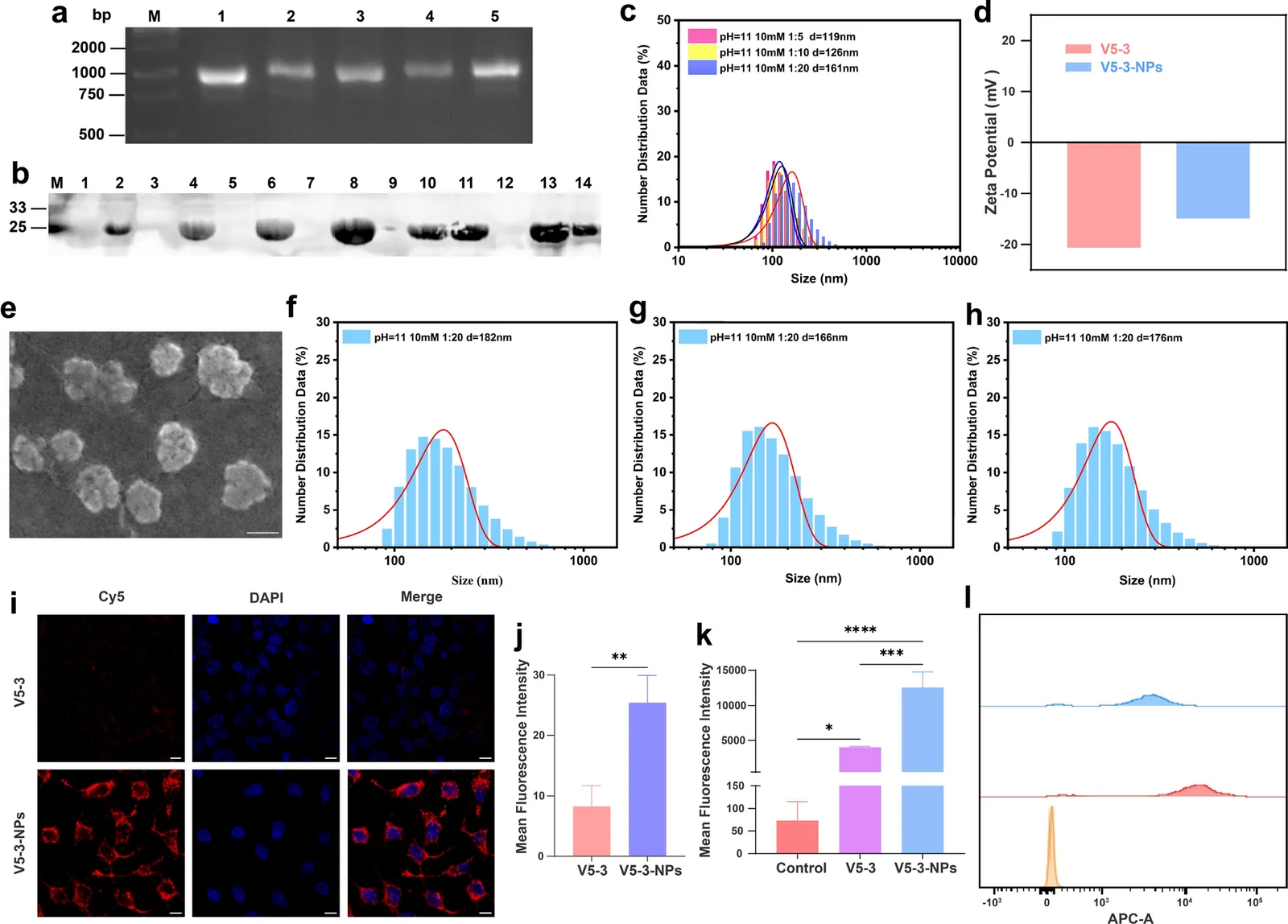
Characterizations of the V5-3 NPs. (**a**) PCR results. Bands at 750 bp indicated successful transformation of the recombinant plasmid into E. coli. (**b**) Western blot results of the induced expanded expression and purification process. M: Marker; 1: Pre-induction; 2: Post-induction; 3: Lysis supernatant; 4: Lysis pellet; 5: TGE washing supernatant; 6:TGE washing pellet; 7: 3 M first washing supernatant; 8: 3 M first washing pellet; 9: 3 M second washing supernatant; 10: 3 M second washing pellet; 11: Binding dissolution supernatant; 12: Flow-through; 13: First elution; 14: Second elution. (**c**) Size distribution of V5-3-NPs prepared at different V5-3:DM ratios (1:5, 1:10, 1:20) in 10 mM solution at pH 11. (**d**) Zeta potential of V5-3-NPs. (**e**) SEM characterization of V5-3-NPs (Scale bar: 200 nm). (**f–h**) Stability assessment of reconstituted freeze-dried V5-3-NPs stored at 4 °C for 1 day (**f**), 3 days (**g**), and 7 days (**h**). (**i, j**) Representative confocal images (**i**) and corresponding quantitative analysis (**j**) of HCE-T cells incubated with V5-3-Cy5 or V5-3-NPs-Cy5 for 1 h (Scale bar: 10 μm, magnification 100 ×). Blue: Hoechst 33,342; Red: Cy5. (**k, l**) Flow cytometry histograms (**l**) and quantification of mean fluorescence intensity (MFI) (**k**) of HCE-T cells incubated with V5-3-Cy5 or V5-3-NPs-Cy5. Data are presented as means ± standard deviation (SD). n ≥ 3. *P* < 0.05 (*), *P* < 0.01 (**), *P* < 0.001 (***), *P* < 0.0001 (****)

The recombinant plasmid encoding V5-3 was successfully constructed and transfected into *E. coli*, as confirmed by polymerase chain reaction (PCR) analysis (Fig. 3a). Following induction of *E. coli*, the expressed V5-3 protein was purified to > 98% purity, as verified by sodium dodecylsulfonate-polyacrylamide gel electrophoresis (SDS-PAGE) analysis and Western blot analysis (Figures S1f and 3b). The purified recombinant protein displayed a molecular weight ranging from approximately 25 kDa to 35 kDa. To further verify the protein, Matrix-assisted laser desorption/ionization time-of-flight mass spectrometry (MALDI-TOF MS) was employed. A distinct peak at m/z = 32,817.48 confirmed the molecular weight of the V5-3 as approximately 32.817 kDa (Figure S1g).

The biological activity of the generated bispecific antibody V5-3 was evaluated using ELISA. The results showed a significant increase in OD values in the experimental group compared with the control group. This finding confirmed that the V5-3 specifically binds human and murine VEGF or TNF-α with high affinity. Thus, V5-3 appears to be a promising candidate for anti-inflammatory and anti-angiogenic therapies against uveitis. Furthermore, affinity comparisons showed that V5-3 exhibits higher binding capacity for human antigens than for murine antigens (Figures S1h, i).

### Preparation and characterization of bispecific antibody eyedrops

To fabricate the nano-antibody eyedrops, we synthesized a DM, DM-6C. DM-6C, with a long carbon chain and a positive guanidine motif, can self-assemble with V5-3 to form nanoparticles (V5-3-NPs) through intermolecular hydrogen bonds and hydrophobic interactions. We first optimized the formulation of V5-3-NPs by varying the DM-to-V5-3 ratios (1:5, 1:10, 1:20, 1:30, 1:40, and 1:50) and pH conditions (9, 10, and 11). Dynamic Light Scattering (DLS) measurements were employed to evaluate particle size and distribution uniformity. DLS analysis showed that at pH 11, the NPs displayed improved uniformity and monodispersity, with particle dispersion index (PDI) below 0.3, indicating that the particle size distribution is uniform. Based on these results, V5-3-NPs were synthesized under optimized conditions: pH 11, a V5-3 concentration of 10 mM, and a DM-to-V5-3 ratio of 1:20 (Fig. 3c). Following ultrafiltration and lyophilization, V5-3-NPs exhibited a transparent, gel-like appearance, with a pH value of 7**–**9 upon reconstitution. Considering the delicate ocular environment, the V5-3-NPs eyedrops should remain within the ophthalmic pH range of 5**–**9 to ensure safe ocular administration. Zeta potential of V5-3 was -20.6 mV, whereas that of V5-3-NPs increased to -14.9 mV (Fig. 3d). This implied that the DM-6C formed a stable complex with the V5-3 via electrostatic interactions, in which its cationic groups partially neutralize the antibody’s negative charge. Furthermore, reconstituted V5-3-NPs were characterized by scanning electron microscopy (SEM). As shown in Fig. 3e, V5-3-NPs displayed an irregular spherical morphology with a relatively uniform size of ~ 200 nm, consistent with the particle size determined by DLS. All these results confirmed the successful preparation of V5-3-NPs. The reconstituted V5-3-NPs were stored at 4 °C for 1, 3, and 7 days. Particle size remained stable over this period, with no significant changes detected by DLS, indicating satisfactory stability (Fig. 3f–h).

To study the cellular uptake, V5-3 was labeled with Cy5 (V5-3-Cy5), then mixed with DM-6C to generate nanoparticles (V5-3-NPs-Cy5). Thereafter, human corneal epithelial (HCE‑T) cells were co-incubated with V5-3-NPs-Cy5 or V5-3-Cy5 for 1 h, and quantified via confocal laser scanning microscopy (CLSM) (Fig. 3i, j) and flow cytometry (Figs. 3k, l). Results showed that the red fluorescence intensity of V5-3-NPs-Cy5-treated cells was significantly higher than that of V5-3-Cy5 (*P* < 0.0001), indicating superior cellular uptake.

### Anti-inflammatory and anti-neovascular efficacy of bispecific antibody eyedrops in microglia cells

Microglia, the primary resident immune cells in the central nervous system and retina, initiate uveitis by activating inflammatory pathways and regulating immune infiltration and tissue damage [20]. Therefore, modulating microglial activation is a key therapeutic strategy for uveitis. Human microglial cells (HMC3) and murine microglial cells (BV2) are commonly used cell lines for studying microglial function, as they effectively mimic microglial activation and produce various inflammatory mediators under relevant conditions. Therefore, we selected them as our research models.

**Fig. 4:**
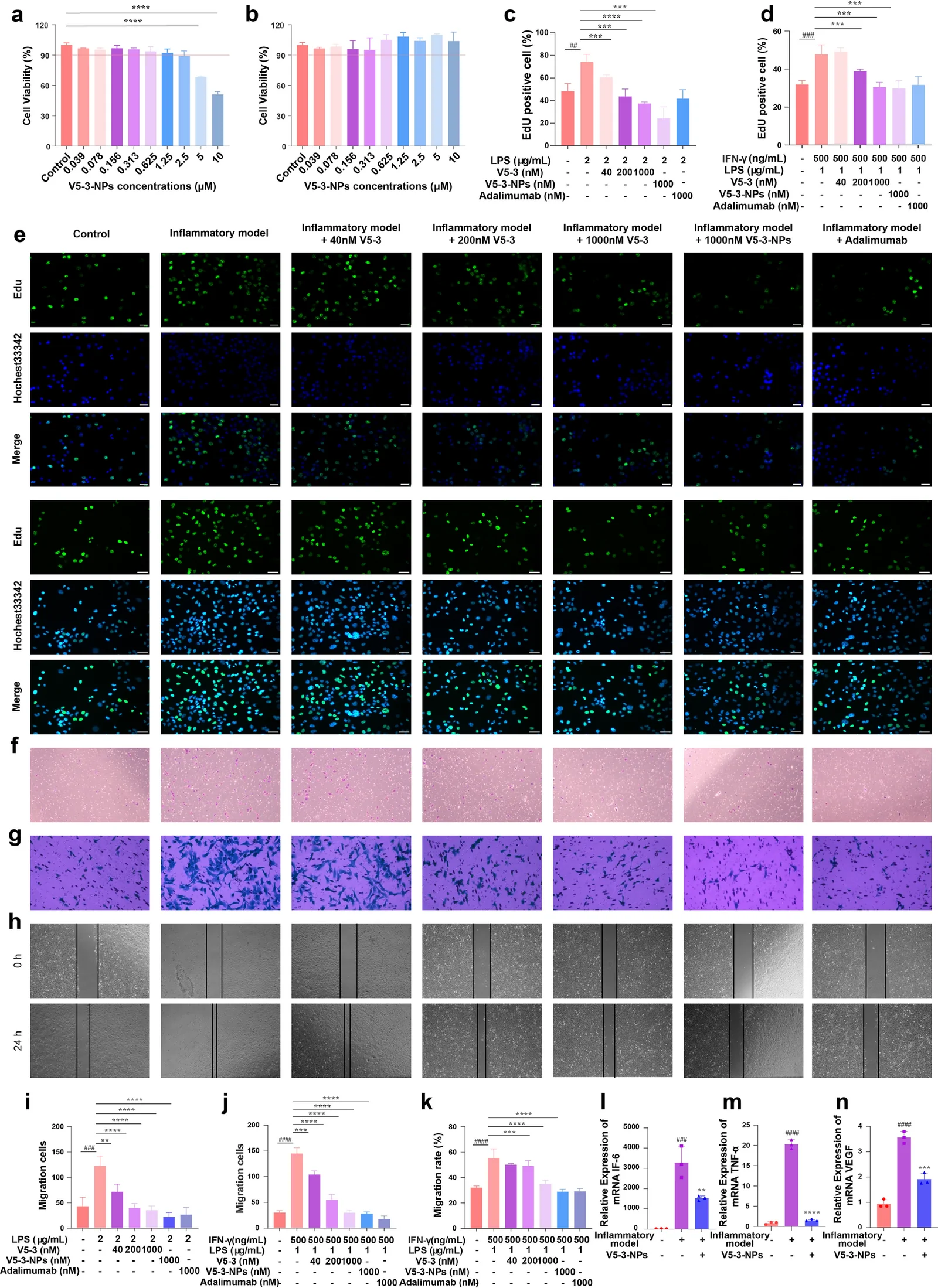
V5-3-NPs suppressed microglial proliferation and migration under inflammatory stimulation. (**a**–**b**) CCK-8 assays measuring the viability of BV2 (**a**) and HMC3 (**b**) cells. (**c**–**e**) EdU assays and corresponding quantitative analyses of BV2 (**c** and **e**, top panels) and HMC3 (**d** and **e**, bottom panels) cells. Green: Alexa Fluor 488 (proliferating cells); Blue: Hoechst 33,342 (nuclei) (Scale bar: 20 μm). (**f-g**) and (**i-j**) Representative Transwell assay images (**f, g**) and corresponding quantitative analyses (**i, j**) of BV2 (**f, i**) and HMC3 (**g, j**) cells (Scale bar: 50 μm). (**h**) and (**k**) Representative scratch wound assay images (**h**) and quantitative analysis (**k**) of HMC3 cells (Scale bar: 50 μm). (**l**–**m**) qRT-PCR analysis of mRNA expression of TNF-α (**l**), IL-6 (m), VEGF (**n**) in HMC3 cells treated with V5-3-NPs, with GAPDH as internal control. Data are presented as means ± SD. n ≥ 3. *P* < 0.01 vs. control (##), *P* < 0.001 vs control (###), *P* < 0.0001 vs control (####). ns, nonsignificant, *P* < 0.01 (**), *P* < 0.001 (***), *P* < 0.0001 (****)

First, the cell counting kit-8 (CCK-8) assay was performed to evaluate the cytotoxicity of V5-3-NPs. BV2 and HMC3 cells were incubated with V5-3-NPs for 24 h over a concentration range of 0.04**–**10 μM. As shown in Fig. 4a and b, BV2 cells maintained above 90% viability with V5-3-NPs concentrations below 1.25 μM, while HMC3 cells exhibited no obvious cytotoxicity across a range of 0**–**10 μM.

Next, we evaluated the anti-proliferation and migration activity of V5-3-NPs on two inflammatory cell models. BV2 cells were first stimulated with 2 μg/mL lipopolysaccharide (LPS) for 24 h, and then treated with V5-3, V5-3-NPs, or Adalimumab (positive control), respectively. 5-ethynyl-2’-deoxyuridine (EdU) staining showed that LPS markedly promoted cell proliferation (Fig. 4c and e), resulting in a 1.8-fold increase compared with the untreated control group (74.33 ± 6.66%, *P* < 0.01), as evidenced by the strong green fluorescence in the LPS-stimulated group (Inflammatory model). Further analysis revealed that the treatment with 1000 nM V5-3-NPs reduced the proliferation rate to 24.33 ± 10.12% (*P* < 0.0001), whereas 1000 nM V5-3 and Adalimumab lowered it to 37.33 ± 1.53% and 41.67 ± 8.08%, respectively (*P* < 0.001). In the subsequent Transwell assay, LPS-stimulated BV2 cells showed a 2.84-fold increase in migration compared with the control group, as confirmed by crystal violet staining (Fig. 4f and i). Treatment with 1000 nM V5-3-NPs reduced migration to 22 ± 7.35 cells (*P* < 0.0001), while V5-3 and Adalimumab suppressed it to 35.33 ± 8.39 and 26.67 ± 11.56, respectively (*P* < 0.0001). These results indicated that both V5-3 and V5-3-NPs effectively inhibited LPS-stimulated proliferation and migration of BV2 cells, with V5-3-NPs showing superior efficacy.

HMC3 cells were then stimulated with 1 μg/mL LPS and 500 ng/mL interferon-γ (IFN-γ) for 24 h, followed by treatment with V5-3, V5-3-NPs, or Adalimumab for an additional 24 h. EdU staining revealed that LPS and IFN-γ stimulation markedly increased green fluorescence in HMC3 cells, indicating enhanced proliferation with a rate of 47.67 ± 4.03% (*P* < 0.001). Both V5-3 and V5-3-NPs exhibited comparable inhibitory effects, with 1000 nM V5-3 reducing proliferation to 30.33 ± 2.05% (*P* < 0.001) and 1000 nM V5-3-NPs decreasing it to 29.67 ± 3.40% (*P* < 0.001). In contrast, Adalimumab treatment exhibited a slightly weaker inhibitory effect (31.33 ± 3.68%, *P* < 0.001) (Fig. 4d, e). Crystal violet staining and quantitative cell counting yielded consistent results, confirming that LPS stimulation induced a 5.8-fold increase in migrated cells (145 ± 9.27, *P* < 0.0001). V5-3 and Adalimumab reduced the migrated cell count to 30 ± 3.74 (*P* < 0.0001) and 28 ± 2.83 (*P* < 0.0001), respectively, while V5-3-NPs demonstrated the greatest inhibitory effect (17.67 ± 5.25, *P* < *0.0001*) (Fig. 4g and j). The scratch wound healing assay revealed that LPS/IFN-γ stimulation markedly accelerated HMC3 cell migration, resulting in 55.27 ± 6.01% wound closure after 24 h (*P* < 0.0001). Treatment with 1000 nM V5-3 reduced migration to 34.87 ± 2.45% (*P* < 0.001), while Adalimumab showed slightly stronger inhibition, achieving 29.13 ± 1.86% closure (*P* < 0.0001). V5-3-NPs at 1000 nM inhibited migration most effectively, reducing wound closure to 28.9 ± 1.47% (*P* < 0.0001) (Fig. 4h and k). Collectively, these findings demonstrated that both V5-3 and V5-3-NPs effectively inhibited inflammation-induced proliferation and migration of HMC3 cells.

To investigate the regulatory effects of V5-3 and V5-3-NPs on inflammation, the effect of V5-3-NPs on the mRNA expression of TNF-α, IL-6, and VEGF in HMC3 cells was further examined using quantitative real-time PCR (qRT-PCR). Results showed that inflammatory stimulation markedly increased TNF-α, IL-6, and VEGF expression in HMC3 cells. Conversely, V5-3-NPs treatment significantly reduced the mRNA expression of these proinflammatory cytokines (Fig. 4l–n). These findings indicated that V5-3-NPs exerted potent anti-inflammatory and anti-angiogenic effects in LPS-stimulated microglia.

### Therapeutic effect of bispecific antibody eyedrops in EAU rat models

**Fig. 5:**
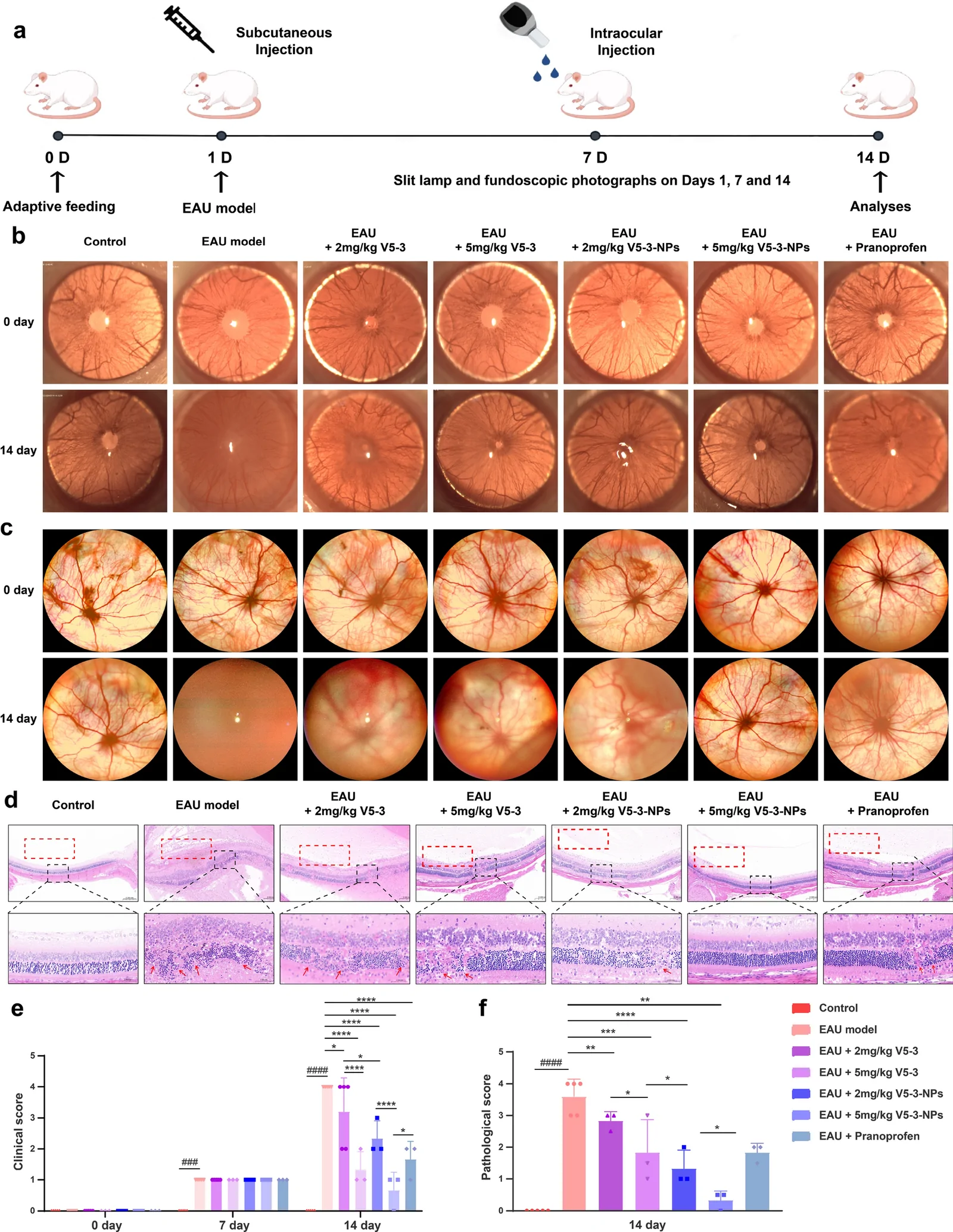
V5-3-NPs ameliorated both clinical symptoms and pathological manifestations in EAU models. (**a**) Treatment timeline. (**b**) Representative slit lamp images (**b**) and corresponding clinical scores (**e**) on Days 1,7 and 14 post-induction. (**c**) Fundoscopic examinations on Days 1,7 and 14 post-induction. (**d**) Representative H&E-stained retinal histopathology images at Day 14 post-induction. (**e**) Clinical scores according to slit lamp images (**b**) on Days 1,7 and 14 post-induction. (**f**) Histopathological scores according to H&E images (**d**) at Day 14 post-induction. Red boxes: inflammatory cell infiltration. Black boxes: magnified regions from the left panels showing retinal folds and optic cell damage. Data are expressed as means ± SD. n = 5. *P* < 0.001 vs control (####). ns, nonsignificant, *P* < 0.05 (*), *P* < 0.01 (**), *P* < 0.001 (***), *P* < 0.0001 (****)

The EAU model was established as shown in Fig. 5a. The EAU model was induced by subcutaneous injection of an immunogenic emulsion, followed by randomization and treatment initiation seven days later. Eyedrops were then topically administered for an additional seven consecutive days. After model induction and drug administration, the rats were examined using slit-lamp microscopy and fundoscopy. Uveitis was characterized by iris hyperemia, pupillary synechiae, and anterior chamber inflammation with cellular infiltration and protein flare. Slit-lamp examination revealed that, compared with the control group, the model group developed typical uveitic features (Fig. 5b). At Day 7, iris vessel dilation was observed, and progressed to anterior chamber exudation and pupillary synechiae formation by Day 14, confirming successful EAU induction. Therapeutic efficacy was evaluated for V5-3 and V5-3-NPs eyedrops, with the clinically approved drug, pranoprofen, as a positive control. Compared with the EAU model group, rats treated with either V5-3 or V5-3-NPs eyedrops showed a significant reduction in iris vascular dilation, complete resolution of iris hyperemia, and alleviation of inflammatory responses, demonstrating their promising therapeutic potential against uveitis (Fig. 5b). Fundoscopic examination further revealed severe retinal detachment in the model group. After treatment, gradual restoration of retinal structure was observed (Fig. 5c). Clinical scoring indicated that V5-3-NPs (5 mg/kg) achieved the greatest reduction in clinical scores, outperforming both pranoprofen and V5-3 treatments (Fig. 5e).

After treatments, the rats were euthanized, and ocular tissues were collected for histological sectioning and hematoxylin and eosin (H&E) staining. Histological analysis revealed distinct pathological changes in the EAU model group, including disorganized retinal architecture, pronounced retinal folding, and substantial inflammatory cell infiltration. In contrast, all treated groups showed significant improvement in these pathological alterations. Importantly, the V5-3-NPs (5 mg/kg) group demonstrated remarkable therapeutic efficacy, achieving near-complete histological restoration, with no appreciable difference from the control group (Fig. 5d and f). Collectively, these findings suggest that V5-3-NPs provide superior pharmacological efficacy and improved bioavailability compared with the partial effect observed with V5-3.

**Fig. 6:**
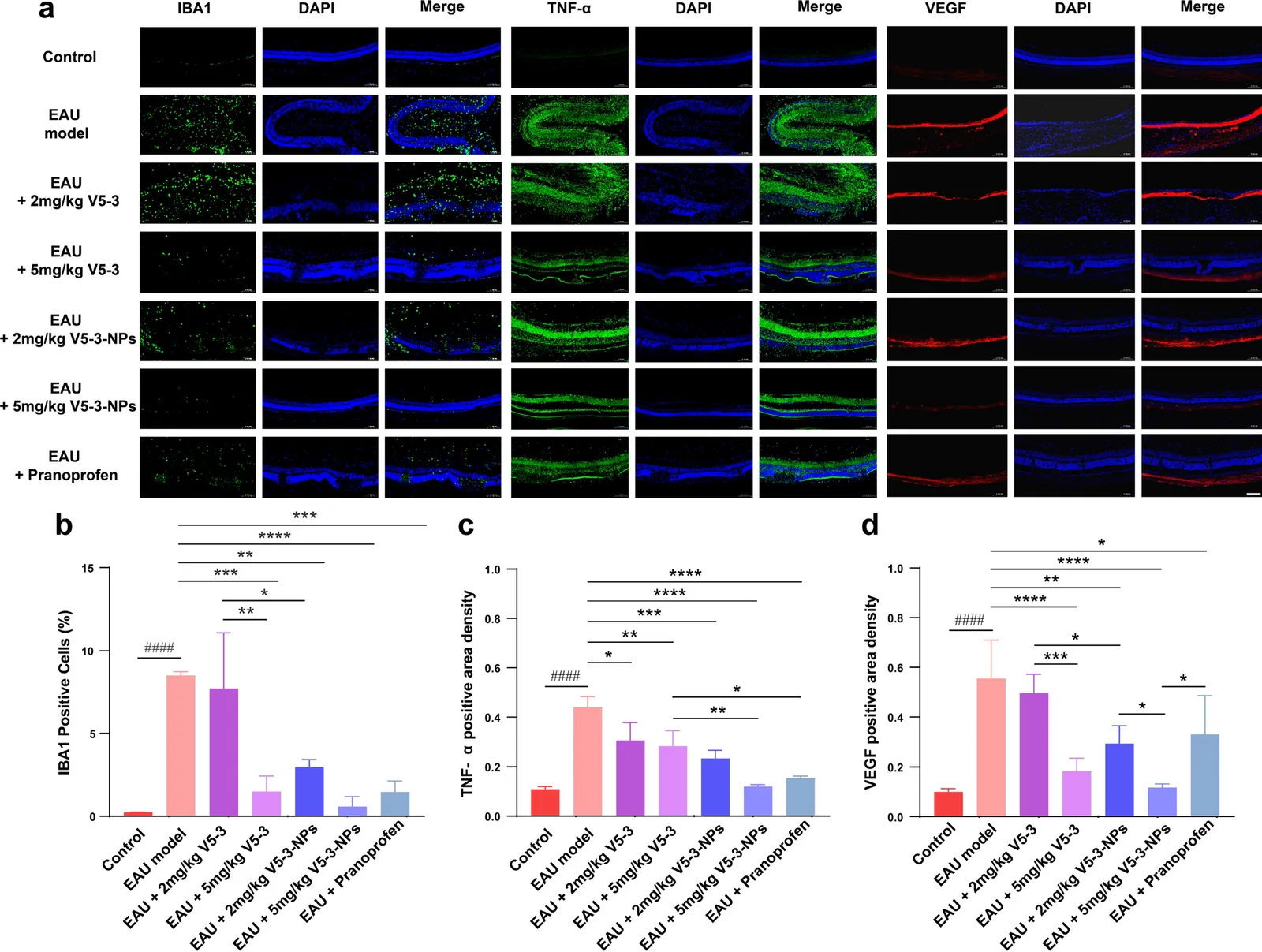
V5-3-NPs attenuated the expression of IBA1, TNF-α, and VEGF in EAU models. (**a-d**) Representative immunofluorescence images of retinal tissues from EAU rats across treatment groups (**a**), and corresponding quantitative analyses (**b-d**). Samples were stained for IBA1 (Green), TNF-α (Green), VEGF (Red) and DAPI (Blue). Data are expressed as means ± SD. Scale bar: 100 μm. n = 5. ns, nonsignificant, *P* < 0.001 vs control (####), *P* < 0.05 (*), *P* < 0.01 (**), *P* < 0.001 (***), *P* < 0.0001 (****)

TNF-α and VEGF are key mediators in the pathogenesis of uveitis. VEGF promotes disease progression by inducing pathological neovascularization and increasing vascular permeability, ultimately leading to exudative retinopathy and visual impairment [21]. As a major regulator of intraocular inflammation, TNF-α contributes to uveitis by triggering vascular inflammation and disrupting the blood-retinal barrier [22]. In EAU models, ionized calcium-binding adapter molecule 1 (IBA1), a microglia/macrophage-specific regulatory protein, exhibited a strong correlation with inflammatory progression in retinal and choroidal tissues. IBA1 facilitates inflammatory cell infiltration into uveal tissues via regulating actin polymerization, chemokine secretion, and phagocytic activity [23, 24]. Clinical studies have revealed significantly elevated numbers of IBA1-positive cells in the vitreous humor and retinal tissues of uveitis patients, which correlate strongly with disease activity [25]. These findings identify IBA1 as a potential biomarker for disease severity and prognosis in uveitis. Building on the established roles of IBA1, TNF-α, and VEGF in uveitis, immunofluorescence analysis showed marked upregulation of these markers in retinal tissues of EAU rats compared with controls. Among all interventions, V5-3-NPs at 5 mg/kg produced the strongest inhibitory effect on these pathogenic markers **(**Fig. 6a–d**)**.

### Safety assessment of V5-3 and V5-3-NPs

Having identified 5 mg/kg as the optimal therapeutic dose based on the preceding efficacy studies, we next evaluated the safety profile of V5-3-NPs at this concentration. Given that intraocular pressure (IOP) homeostasis is essential for preserving retinal ganglion cell function, we measured IOP by non-contact tonometry using a Tonolab tonometer after 1 week of consecutive treatment with V5-3 and V5-3-NPs. As shown in Figure S2b, all treatment groups maintained stable IOP within the normal range (9–11 mmHg), without significant differences. These results suggest that neither V5-3 nor V5-3-NPs were likely to induce IOP fluctuations. Representative slit-lamp and fundus images revealed no adverse effects— including hyphema, corneal opacity, intraocular inflammation, or neovascularization— in treated groups or controls from baseline to Day 7 (Figure S2a). One week after treatment, the animals were euthanized for histopathological evaluation. H&E staining of ocular tissues showed intact corneal structure without pathological changes, such as thickening, disorganization, neovascularization, or inflammatory cell infiltration. The retinal layers remained well-preserved, with no evidence of folding, disruption, or exudation (Figure S2c). Systemic evaluation of major organs, including the heart, liver, spleen, lung, and kidney, revealed no detectable pathological alterations (Figure S2d). Collectively, these results indicated favorable biosafety and biocompatibility of both V5-3 and V5-3-NPs.

## Discussion

The uvea, characterized by its rich vascularization and abundance of immunologically active cells, is highly susceptible to inflammatory responses. Uveitis can induce significant intraocular tissue damage, leading to symptomatic discomfort and complications that may progress to severe visual impairment or permanent vision loss [26]. VEGF and TNF-α are both central mediators in uveitis pathogenesis, but monotherapies targeting a single pathway fail to fully control disease progression. Besides, ocular drug administration is restricted by complex anatomical and physiological barriers, leading to low bioavailability, considerable side effects, and the need for invasive administration [27]. These challenges highlight the urgent need for innovative drug delivery strategies. This study presents a dual-targeted nanotherapeutic strategy for uveitis treatment. By combining VEGF/TNF-α bispecific antibody engineering with dithiolane-based nanoparticle delivery, we achieved simultaneous suppression of pathological angiogenesis and intraocular inflammation through non-invasive administration.

Through bioinformatic analysis, we identified and prioritized 155 uveitis-associated genes from the DisGeNET and GeneCards databases. Construction of a PPI network using STRING and Cytoscape identified TNF, VEGFA, and IL-6 as hub genes in uveitis pathogenesis, while DEGs analysis of the GSE66936 dataset revealed enrichment in immune and inflammatory signaling pathways. TNF-α and VEGF are key drivers of uveitis onset and progression. TNF-α is a central upstream inflammatory cytokine that activates multiple downstream signaling cascades, including NF-κB and MAPK pathways, thereby amplifying inflammatory cytokine production (including IL-6), leukocyte recruitment, and retinal tissue damage [28, 29]. VEGF, a pro-angiogenic cytokine upregulated in inflamed microenvironments, promotes pathological neovascularization. Moreover, by binding to VEGFR-2, VEGF activates the MAPK/ERK pathway, thereby enhancing inflammatory cell activation and migration [30]. These two signaling modules are not parallel but rather converge at multiple nodes; for example, NF‑κB can directly enhance VEGF transcription [31], while VEGF‑induced ERK signaling can potentiate TNF‑α‑driven inflammatory gene expression, creating a self‑reinforcing loop that perpetuates uveitic inflammation and pathological neovascularization [32]. Therefore, simultaneous inhibition of VEGF and TNF-α may interrupt both inflammatory amplification and neovascularization pathways, potentially contributing to more comprehensive suppression of ocular inflammation than single-target intervention alone.

We engineered a VEGF/TNF-α bispecific antibody— V5-3— to simultaneously inhibit angiogenesis and inflammation. SDS-PAGE, WB, MALDI-TOF MS, and ELISA confirmed effective binding to both human and murine VEGF and TNF-α, with high affinity for human proteins. These results validated V5-3 as distinct from conventional single-target antibodies, simultaneously targeting TNF-α and VEGF and integrating both anti-inflammatory and anti-angiogenic activities. Then, we optimized the assembly ratios and solution pH for V5-3-NPs formation. This strategy significantly enhanced cellular uptake in HCE-T models and confirmed structural stability via DLS and SEM. Similar to our previously reported dithiolane-based ocular nanoformulations, the present V5-3-NPs are expected to undergo intracellular GSH-responsive disassembly, which may facilitate antibody release after cellular internalization [33].

In uveitis, microglia shift from the M2 to the M1 phenotype, amplifying inflammatory cascades and worsening retinal damage [34]. In BV2 and HMC3 uveitis models, both V5-3 and V5-3-NPs suppressed inflammation-induced proliferation, migration, and cytokine secretion, while exerting potent anti-inflammatory and anti-angiogenic activities. V5-3-NPs outperformed V5-3, showing stronger inhibition of key inflammatory mediators, including TNF-α, IL-6, and VEGF. In EAU rat models, V5-3-NPs outperformed V5-3 by significantly reducing clinical symptoms and histopathological damage compared with positive controls. V5-3-NPs reduced iris hyperemia, anterior chamber exudation, pupillary adhesion, and retinal detachment, while downregulating retinal TNF-α, VEGF, and IBA1 expression. The superior efficacy of V5-3-NPs is probably attributed to the simultaneous inhibition of VEGF and TNF-α [15], which disrupts the interconnected NF-κB and MAPK signaling axes central to uveitis pathogenesis, suppressing upstream inflammation and downstream angiogenesis [18]. Notably, the markedly greater efficacy of V5-3-NPs compared with free V5-3 eyedrops suggests improved intraocular bioavailability, likely reflecting enhanced corneal penetration. In our previous study, similar DMs were employed to deliver anti‑VEGF antibody or siRNA, and fluorescently labeled conjugates confirmed efficient corneal penetration and intraocular distribution following topical instillation [33, 35]. Mechanistic investigations further revealed that the DMs facilitate transcorneal delivery via the thiol‑mediated transport pathway. Cellular uptake assays in HCE‑T cells further corroborated this notion, demonstrating that DM markedly enhanced antibody internalization. Overall, the observed improvements of V5-3-NPs were accounted for the synergistic mechanism between the VEGF/TNF-α bispecific antibody and the nanocarrier-mediated ocular tissue penetration [36].

In clinical practice, corticosteroids remain the first-line topical therapy for uveitis; however, their long-term use is limited by well-established adverse effects, including ocular hypertension, cataract formation, and increased susceptibility to infection [37]. Notably, our safety evaluation demonstrated that V5-3-NPs did not elevate intraocular pressure or cause systemic toxicity throughout the treatment period. When compared with pranoprofen, a widely used nonsteroidal anti-inflammatory drug (NSAID) eye drop, V5-3-NPs exhibited markedly superior therapeutic efficacy. These findings highlight the clinical potential of V5-3-NPs as a convenient and noninvasive topical therapy for long-term uveitis management, with robust therapeutic efficacy and favorable safety profiles.

Despite these promising results, several limitations of the present study should be acknowledged, as they also point to important directions for future investigation. First, although the current formulation showed satisfactory short-term stability upon reconstitution, detailed long-term stability evaluation and quantitative release kinetics of V5-3-NPs remain important future directions for further translational development, particularly to meet the 1–2 month shelf-life requirements of clinical eye drop products. Second, while the use of adalimumab as a comparator provided partial insight, a more definitive comparison between dual-target and single-target strategies—ideally including an anti-VEGF monospecific antibody arm—is needed to fully dissect the individual contributions of TNF-α and VEGF blockade and to quantify the synergistic benefit of the bispecific design. Third, future head-to-head comparisons with topical corticosteroids (e.g., dexamethasone or prednisolone acetate) will be essential to benchmark the anti-inflammatory efficacy, intraocular pressure safety, and cataractogenic risk of V5-3-NPs against the current clinical gold standard. Beyond these priorities, quantitative ocular pharmacokinetic profiling, evaluation of therapeutic dosing regimens initiated at the peak of inflammation, and long-term ocular and systemic safety studies in larger animal models will be required to advance this bispecific nano-antibody eye drop toward clinical application.

## Conclusion

In summary, our study identified hub genes of uveitis through bioinformatic analysis and laid the groundwork for future research. We engineered a VEGF/TNF-α bispecific antibody and encapsulated it into DM-based nanoparticles. This bispecific antibody inhibited dual pathways by simultaneously suppressing VEGF-driven angiogenesis and TNF-α-mediated inflammatory cascades, thereby ameliorating both clinical manifestations and histopathological damage. The dithiolane nanocarrier further enhanced the therapeutic efficacy by providing superior tissue penetration and cellular uptake, thereby improving bioavailability while maintaining safety at therapeutic concentrations. Collectively, these findings position the bispecific antibody eyedrops as a promising candidate for further development in uveitis therapy.

## Methods

### Bioinformatics analysis

Uveitis-associated genes were retrieved from the DisGeNET and GeneCards databases, with core targets identified through Venn diagram analysis. PPI network was constructed using STRING database, visualized and analyzed in Cytoscape 3.9.1 with CytoHubba plugin for node degree assessment (degree ≥ 1).

GEO datasets from uveitis patients and healthy controls were analyzed using the limma package in R to identify DEGs, with thresholds of |Log_2_FC|> 1.5 and *P* < 0.05. Gene annotation was conducted using the org.Hs.eg.db package to establish the reference background set, followed by GO and KEGG enrichment analyses via cluster Profiler R package, retaining terms meeting gene set size (5**–**5000) and thresholds (*P* < 0.05, FDR < 0.1).

The 3D structure of V5-3 was predicted through homology modeling using SWISS-MODEL. This model, along with TNF-α and VEGFA protein PDB files obtained from the RCSB Protein Data Bank, was imported into the HawkDock server (http://cadd.zju.edu.cn/hawkdock/) for docking simulations to predict their binding poses [38]. The resulting complexes were subsequently visualized using PyMOL v2.5.0.

### Synthesis and identification of V5-3

The plasmid encoding a fusion gene of VEGF and TNF-α antibody fragment was transformed into competent E. coli BL21 (DE3) cells (Quanshijin Biotech, Beijing) using the heat-shock method. Recombinant clones were screened by PCR amplification before agarose gel electrophoresis, with T7 promoter primer and T7 Terminator primer. Following Isopropyl-β-D-thiogalactopyranoside (IPTG) induction, positive constructs were subsequently heat-denatured (98 °C, 10 min), resuspended in TGE buffer, and lysed by ice-bath sonication. The His-tagged recombinant protein was incubated with pre-equilibrated magnetic beads (Beaver Bioengineering Company, Suzhou, China, Cat#70,501–5) for purification. The purified protein was then subjected to urea-gradient dialysis for desalting, filtered through a 0.22 μm membrane, and quantified by BCA assay before use. The purified recombinant protein was subsequently resolved on 15% SDS-PAGE and characterized by MALDI-TOF MS to confirm molecular weight and protein identification.

### Western Blot (WB)

The separated proteins were transferred onto polyvinylidene fluoride (PVDF) membranes for immunoblotting analysis. After blocking in 5% skim milk for 1 h, the membrane was incubated with primary antibodies (HRP Anti-6X His tag, 1:1000; Abcam, Cat#ab1187) at 4 °C overnight, followed by a 2-h incubation with corresponding horseradish peroxidase (HRP)-conjugated secondary antibodies (HRP-labeled rabbit anti-mouse, 1:10,000; Beyotime, China) at room temperature. Protein bands were visualized using Ultra High Sensitivity ECL Kit (Glpbio, China, Cat#GK10008). The grayscale values of the protein bands were then quantified by Image J.

### MALDI-TOF MS

After desalting by dialysis, the protein solution was diluted to 0.1**–**1 μg/mL with ddH_2_O. A saturated matrix solution was prepared by mixing 1% trifluoroacetic acid (TFA) in ddH_2_O with an equal volume of acetonitrile, followed by the addition of sinapic acid. The resulting solution was used as a matrix and mixed with the protein sample at a 1:1 ratio. Then 1 μL of the protein-matrix mixture was spotted onto the MALDI-TOF target plate. After air-drying to form crystals, the target plate was loaded into the mass spectrometer for final spectral acquisition.

### ELISA assay

Each well was coated with 100 μL (50 ng) of VEGF or TNF-α and incubated overnight at 4 °C. The plates were then blocked with 5% Bovine Serum Albumin (BSA) at 37 °C for 2 h after being washed 3 times with PBST (PBS containing 0.05% Tween 20). V5-3 was then diluted to 0.5 mg/mL with 2% BSA before use, and each well received 100 μL of the dilute, followed by a 1-h incubation at room temperature. Subsequently, the plates were sequentially incubated with primary antibody (anti-His-Tag mouse mAb, 1:10,000 in 1% BSA; CST, USA) and secondary antibody (1:50,000 in 1% BSA) each for 1 h at 37 °C. Finally, each well received 100 μL TMB substrate for color development, with color change monitored every 5 min until the reaction was terminated by stop solution (Solarbio, China, Cat#C1058). The absorbance at 450 nm was measured within 10 min after termination on an ELISA plate reader (BioTek, USA).

BV2 cells were cultured at 3 × 10^5^ cells/well in 6-well plates for 24 h, then treated according to experimental groupings for another 24 h. Cell culture supernatants were finally collected for quantifying the levels of TNF-α and IL-6 with the indicated ELISA kits (Lianke, Hangzhou, China), in line with the manufacturer’s protocols. Absorbance at 450 nm was measured using an ELISA plate reader (BioTek, USA).

### Synthesis of V5-3-NPs

Alphalipoic acid (784 mg, 3.8 mM) and 1,1’-Carbonyldiimidazole (CDI, 812 mg, 5.0 mM) were dissolved in 25 mL anhydrous dichloromethane (DCM), while hexamethylenediamine (2 mL, 30 mmol) was separately prepared in 5 mL anhydrous DCM. The former solution was added dropwise to the latter at 0 °C with 30 min’ stirring at room temperature. The mixture was then washed with brine to remove water-soluble impurities, dried over anhydrous sodium sulfate (Na_2_SO_4_), and concentrated under reduced pressure to obtain a yellow oil (6C thiol intermediate). This intermediate was dissolved in anhydrous DCM under stirring at 50 °C and reacted with equimolar 1H-pyrazole-1-carboxamidine hydrochloride (293 mg, 2 mM) overnight at room temperature. After solvent removal by rotary evaporation, the residue was dissolved in methanol and precipitated with diethylether (Et2O). The precipitate was washed repeatedly with Et2O and dried to obtain pure DM as a yellow solid. V5-3-NPs were then assembled by mixing the resulting DM with V5-3 at a specified ratio, followed by 1-h incubation at 37 °C in the dark.

### Characterization of V5-3-NPs

The prepared V5-3-NPs were dissolved in NaCl solution for zeta potential and size distribution analysis. The sample was loaded into the U-shaped electrophoresis cell. DLS (Nano-ZS, Malvern) measurements were performed with triplicate readings per sample, and the mean value was calculated. Data reliability was confirmed using zeta potential values and PDI criteria. The lyophilized V5-3-NPs were redispersed and stored at 4 °C for 1, 3, and 7 days, followed by hydrodynamic diameter measurement via DLS.

50 μL of suspension was deposited onto anti-slip pretreated glass slides and air-dried at room temperature. The samples were then mounted on conductive carbon tape and sputter-coated with gold using an ion sputter coater for approximately 30 s prior to field emission SEM (SU8200, Hitachi) observation.

### Evaluation of cellular uptake efficiency of V5-3-NPs

V5-3 was Cy5-labeled using the Cy5 Fluorescent Antibody/Protein Labeling Kit (Ruixi, China, Cat# R-SJH-003). V5-3-Cy5 was then combined with the DM at a specified ratio to yield V5-3-NPs-Cy5.

HCE-T cells were cultured at 1 × 10^4^ cells/dish in 35 mm diameter dishes and co-incubated with V5-3-NPs-Cy5 or V5-3-Cy5 for 1 h. The cells were then stained with Hoechst 33,342 (1:1000, Yeasen, China, Cat#C1027) for 15 min at room temperature to label nuclei. Confocal microscopy images were acquired and analyzed for fluorescence intensity using ImageJ.

HCE-T cells were cultured at 1 × 10^5^ cells/dish in 6-well plates for 24 h. The cells were then co-incubated with 10 nM V5-3-NPs-Cy5 or V5-3-Cy5 for 2 h at 37 °C. Cellular fluorescence was subsequently analyzed by flow cytometry (FACS Canto TM II, BD, USA).

### Cell culture and treatment paradigms

BV2 and HMC3 cells were purchased from Procell Life Technology (Wuhan, China) and were routinely cultured in their respective specialized media purchased from Haixing Biotechnology (Suzhou, China, Cat#TCH-G399, Cat#TCM-G718) at 37 °C in a humidified 5% CO2 incubator.

After the confluence rate reached 80**–**90%, inflammatory models were established by treating BV2 cells with 1 μg/mL LPS) for 24 h and HMC3 cells with 1 μg/mL LPS and 500 ng/mL IFN-γ for 24 h. The stimulated cells were then exposed to various concentrations of V5-3, V5-3-NPs, or 1000 nM Adalimumab (positive control) for 24 h according to experimental groupings.

### Cell viability analysis

BV2 and HMC3 cells were seeded at 5 × 10^4^ cells/mL in 96-well plates for 24 h. The cells were then treated with various concentrations of V5-3-NPs, while the control group received an equal volume of PBS buffer for another 24-h culture. Following 10 μL of CCK-8 solution (Meilun, Dalian, China, Cat#MA0218) added to each well, the plates were incubated for 1 h at 37 °C, and the absorbance at 450 nm was immediately measured with an enzyme meter. The results were expressed as the ratio of cell viability.

### EdU-488 cell proliferation assay

BV2 and HMC3 cells were serum-starved for 12 h and seeded in 96-well plates for overnight culture. Their cell proliferation was detected using BeyoClick™ EdU Cell Proliferation Kit with Alexa Fluor 488 (Beyotime, Shanghai, China, Cat#C0071S) according to the manufacturer’s protocols.

### Transwell migration assay

BV2 and HMC3 cells in logarithmic growth phase were resuspended in serum-free medium and seeded into Transwell upper chambers. Drug solutions in 2% Foetal Bovine Serum (FBS)-containing medium were pre-warmed at 37 °C for 30 min, then added to the lower chambers. Following 24-h incubation at 37 °C, migrated cells attached to the lower membrane were fixed with 4% paraformaldehyde (PFA) for 30 min, stained with 0.1% crystal violet for 10 min, and finally imaged under a microscope. Cell migration was quantified by counting cells in 5 randomly selected fields per well.

### Wound healing assay

HMC3 cells were seeded in 6-well plates and cultured to 100% confluence. After starvation for 12 h, scratches were created perpendicular to pre-marked guide lines using sterile pipette tips. Cells were then treated with FBS-free culture medium containing specified drug formulations according to experimental groups and incubated for 24 h. Images were captured at 0 h and 24 h after scratching at five predetermined positions per well. Migration rates were quantified by Image J.

### qRT-PCR

HMC3 cells were cultured at 3 × 10^5^cells/well in 6-well plates for 24 h before group-specific treatments for another 24 h. RNA was then extracted using an RNA isolation kit (Omega, Cat#R6834-02), and reverse-transcribed into cDNA using All-in-One First-Strand Synthesis MasterMix (Herui, Fujian, China, Cat#HRF0182) according to the manufacturer’s protocol. PCR reactions were carried out on the Roche LightCycler 96 System using SYBR Green. The primer sequences are listed in Table 1. Relative quantification of gene expression was calculated using the 2^−ΔΔCt^ method with *GAPDH* as an internal control.

**Table 1:** qRT-PCR primers

Marker	Primer sequences
*GAPDH*	Forward: GGTGTGAACCATGAGAAGTATGA Reverse: GAGTCCTTCCACGATACCAAAG
*TNF-α*	Forward: TAAGCAACAAGACCACCACTTC Reverse: TCTCCAGATTCCAGATGTCAGG
*IL-6*	Forward: ATGCAATAACCACCCCTGAC Reverse: GCGCAGAATGAGATGAGTTGT
*VEGF*	Forward: ATCGAGTACATCTTCAAGCCAT Reverse: GTGAGGTTTGATCCGCATAATC

### Animal preparation and ethical statement

Male Lewis rats (7–8 weeks, 200 g, SPF) were purchased from Beijing Vital River Laboratory Animal Technology Company. All animal experiments were performed in compliance with the relevant laws and approved by the Institutional Animal Care and Use Committee of Fujian Medical University (IACUC FJMU 2024-Y-1428).

### Model induction, treatment grouping, and scoring

The bovine-derived PDSAg peptide (341**–**354, FLGELTSEVATEV) was dissolved in ultrapure water to prepare a 1 mg/mL stock solution. Mycobacterium tuberculosis was mixed with complete Freund’s adjuvant (CFA, Chondrex, USA) by vortexing to generate a 1 mg/mL CFA suspension. The peptide solution and CFA suspension were then emulsified in equal volumes. Under anesthesia, rats received bilateral thigh intramuscular injections (50 μL, 7.5 μg PDSAg each) and a tail-base subcutaneous injection (100 μL), totaling 200 μL (15 μg PDSAg).

Lewis rats were randomly assigned to seven groups (n = 5 each) after EAU model induction. From Day 7 post-modeling, treatments were administered via ocular surface instillation three times daily (one drop per eye per administration) for 7 consecutive days. Pranoprofen eyedrops (Qianshou, Beijing, China) served as the positive control. Groups were designated as follows:

1. Control: no experimental manipulation;
2. EAU model: disease model induced without drug administration;
3. EAU + V5-3 2 mg/kg: V5-3 eyedrops (20 mg/mL) administered after modeling;
4. EAU + V5-3 5 mg/kg: V5-3 eyedrops (50 mg/mL) administered after modeling;
5. EAU + V5-3-NPs 2 mg/kg: V5-3-NPs eyedrops (20 mg/mL) administered after modeling;
6. EAU + V5-3-NPs 5 mg/kg: V5-3-NPs eyedrops (50 mg/mL) administered after modeling;
7. EAU + Pranoprofen: Pranoprofen eyedrops administered after modeling.

The eyedrops were formulated at concentrations calculated based on body weight to ensure consistent therapeutic mass per dose. Topical instillation was performed using a calibrated micropipette to deliver a fixed volume (20 μL per instillation) per eye per administration.

Following EAU model induction and treatments, anterior segment inflammatory manifestations were examined using slit-lamp microscopy, and corresponding clinical scores were graded according to the standard criteria [39].

### Animal sample collection and processing

On Day 14 post-modeling, all experimental animals were euthanized under anesthesia. Their ocular tissues were immediately harvested, rinsed with cold PBS to remove blood contaminants, and subsequently immersion-fixed in FAS fixative for histological processing, including H&E staining and immunohistochemistry (IHC). Histopathological scoring of ocular tissue sections was based on the previously described criteria [39].

### Biocompatibility of V5-3 and V5-3-NPs in rats

Ocular examinations were performed on Day 0 and Day 7 with slit-lamp biomicroscopy and fundoscopy, respectively. At one week post-treatment, IOP was measured noninvasively with a Tonolab rebound tonometer. The mean IOP value for each eye was calculated from three consecutive measurements. One week after treatment, experimental animals were euthanized for histological examination for multiorgan toxicity profiling, with hearts, livers, spleens, lungs, kidneys, and eyes harvested for H&E staining and retinal structural analysis.

### Statistical analysis

Data statistical analyses were performed using the Statistical Package for Social Sciences software (SPSS, V. 26.0, IBM, USA). Normality was assessed by the Shapiro–Wilk test, followed by independent t-tests for normally distributed data or Mann–Whitney U tests for non-parametric data. Homogeneity of variance was verified using Levene’s test, with one-way ANOVA employed for multi-group comparisons. All continuous values were expressed as mean ± SD. *P* < 0.05 was considered statistically significant.

## Supplementary Information

Additional file 1.

## Data Availability

No datasets were generated or analysed during the current study.

## Abbreviations

BP: Biological pathway
BV2: Murine microglial cells
CCK-8: Cell counting kit-8
CC: Cellular components
CDI: Carbonyldiimidazole
Ce-MOFs: Cerium-based metal–organic frameworks
CLSM: Confocal laser scanning microscopy
DCM: Dichloromethane
DE3: E. coli BL21
DEGs: Differentially expressed genes
DLS: Dynamic light scattering
DM: Dithiolane molecule
DSP: Dexamethasone
EAU: Experimental autoimmune uveitis
EdU: 5-Ethynyl-2’-deoxyuridine
ELISA: Enzyme linked immunosorbent assay
Et2O: Diethylether
FBS: Foetal bovine serum
GEO: Gene expression omnibus
GO: Gene ontology
H&E: Hematoxylin and eosin
HMC3: Human microglial cells
HRP: Horseradish peroxidase
IBA1: Ionized calcium-binding adapter molecule 1
IFN-γ: Interferon-γ
IHC: Immunohistochemistry
IOP: Intraocular pressure
KEGG: Kyoto encyclopedia of genes and genomes
LPS: Lipopolysaccharide
MALDI-TOF MS: Matrix-assisted laser desorption/ionization time-of-flight mass spectrometry
MF: Molecular function
MFI: Mean fluorescence intensity
Na_2_SO_4_: Sodium sulfate
NSAID: Nonsteroidal anti-inflammatory drug
PDI: Particle dispersion index
PFA: Paraformaldehyde
PPI: Protein–protein interaction
PVDF: Polyvinylidene fluoride
qRT-PCR: Quantitative real-time polymerase chain reaction
SD: Standard deviation
SDS-PAGE: Sodium dodecylsulfonate-polyacrylamide gel electrophoresis
SEM: Scanning electron microscope
SPSS: Statistical package for social sciences software
STRING: Search tool for the retrieval of interacting genes/proteins
TFA: Trifluoroacetic acid
TNF-α: Tumor necrosis factor alpha
VEGF: Vascular endothelial growth factor
WB: Western blot
