# Supplementary material for: Dual-targeting VEGF and TNF-α with bispecific antibody eyedrops for synergistic treatment of uveitis
Source: J Nanobiotechnology. 2026 Jun 16;24:801. doi: 10.1186/s12951-026-04681-y (PMC13501631; doi:10.1186/s12951-026-04681-y)
Supplement: Supplementary file 1 [file 12951_2026_4681_MOESM1_ESM.docx]

**Supporting Information**

**Dual-Targeting VEGF and TNF-α with Bispecific Antibody Eyedrops for Synergistic Treatment of Uveitis**

Jie Zhou*^#a,e^, Yinqi Chen^#c^, Ruochu Guan^#a^, Wenchao Cai^a^, Zhiqing Lin^a^, Panxiang Rao^a^, Sisi Chen^a^, Penfeng Gao^g^, Qian Yang^a,e^, Ying Wu^a^, Yihua Zhu^c,d^, Biting Zhou^c,d^, Nanwen Zhang^e,f^, Juhua Yang*^a,b^, Xiaole Chen*^a,b^

^a^ Department of Bioengineering and Biopharmaceutics, School of Pharmacy, Fujian Medical University, Fuzhou, Fujian, 350122, China.

^b^ Fujian Key Laboratory of Drug Target Discovery and Structural and Functional Research, School of Pharmacy, Fujian Medical University, Fuzhou, Fujian, 350122, China.

^c^ Department of Ophthalmology, The First Affiliated Hospital of Fujian Medical University, Fuzhou, Fujian, 350000, China.

^d^ National Regional Medical Center, Binhai Campus of the First Affiliated Hospital, Fujian Medical University, Fuzhou, 350000, China.

^e^ Key Laboratory of Natural Medicine Pharmacology in Fujian Province, School of Pharmacy, Fujian Medical University, Fuzhou, Fujian, 350122, China.

^f^ Department of Pharmacology, School of Pharmacy, Fujian Medical University, Fuzhou, Fujian, 350122, China.

^g^ FuZhou Aier Eye Hospital, Fuzhou, Fujian, 350000, China.

Corresponding author:

Prof. Jie Zhou, E-mail: [Jiezhou@fjmu.edu.cn](mailto:Jiezhou@fjmu.edu.cn)

Prof. Juhua Yang, E-mail: [julian_yang@fjmu.edu.cn](mailto:julian_yang@fjmu.edu.cn)

Prof. Xiaole Chen, E-mail: [leochen5139@fjmu.edu.cn](mailto:leochen5139@fjmu.edu.cn)

\# Jie Zhou, Yinqi Chen, and Ruochu Guan contributed equally to this work and are co-first authors.

**Table of Content**

1. Bioinformatic identification of uveitis targets and construction of V5-3. (**Figure S1**)

2. Safety assessment of V5-3 and V5-3-NPs. (**Figure S2**)

**Figure S1.** Bioinformatic identification of uveitis targets and construction of V5-3. (a) Overlapping uveitis-associated genes identified from the DisGeNET and GeneCards databases using Venn diagram analysis. (b) Top 5 hub targets identified by CytoHubba plugin with Betweenness (left panel), BottleNeck (middle panel), and Stress (right panel) algorithms. (c) Intersecting genes across the three algorithms were visualized in a Venn diagram. (d) GO enrichment bubble plots illustrating biological processes (upper panel), cellular components (middle panel), and molecular functions (bottom panel) associated with DEGs. In these plots, the x-axis represents the gene ratio and the y-axis denotes GO terms. Circle color reflects the *P*-value, while circle size corresponds to the number of gene clusters. (e) Heatmap of uveitis-associated DEGs expression profiles. Red: up-regulated genes, Blue: down-regulated genes. Color intensity reflects expression magnitude. (f) SDS-PAGE results of induced expanded expression and purification process. M: Marker; 1: Pre-induction; 2: Post-induction; 3: Lysis supernatant; 4: Lysis pellet; 5: TGE washing supernatant; 6:TGE washing pellet; 7: 3M first washing supernatant; 8: 3M first washing pellet; 9: 3M second washing supernatant; 10: 3M second washing pellet; 11: Binding dissolution supernatant; 12: Flow-through; 13: First elution; 14: Second elution. (g) MALDI-TOF MS analysis of V5-3. (h, i) ELISA assay for binding activity to human-derived (h) and mouse-derived (i) VEGF and TNF-α (n ≥ 3). Data are expressed as means ± SD. *** *P < 0.001* , **** *P < 0.0001.*

**Figure S2.** Safety assessment of V5-3 and V5-3-NPs. (a) Representative slit lamp (left panels) and fundoscopic (right panels) images at one week post-administration. (b) IOP measurements at one week post-administration. (c) H&E staining of ocular tissues. (d) H&E staining of major organs, including the heart, liver, spleen, lung, and kidney. Data are expressed as means ± SD. n = 5. *P* < 0.001 vs control (####). ns, nonsignificant, *P* < 0.1 (*), *P* < 0.01 (**), *P* < 0.001 (***), *P* < 0.0001 (****).
